# Exploring Antimicrobial Resistance in Bacteria from Fecal Samples of Insectivorous Bats: A Preliminary Study

**DOI:** 10.3390/vetsci12060516

**Published:** 2025-05-25

**Authors:** Santina Di Bella, Delia Gambino, Maria Foti, Bianca Maria Orlandella, Vittorio Fisichella, Francesca Gucciardi, Francesco Mira, Rosario Grasso, Maria Teresa Spena, Giuseppa Purpari, Annalisa Guercio

**Affiliations:** 1 Istituto Zooprofilattico Sperimentale della Sicilia, 90129 Palermo, Italy; santina.dibella@izssicilia.it (S.D.B.); francesco.mira@izssicilia.it (F.M.); giuseppa.purpari@izssicilia.it (G.P.);; 2 Dipartimento di Scienze Veterinarie, Università degli Studi di Messina, 98168 Messina, Italy; 3 Dipartimento di Scienze Biologiche, Geologiche e Ambientali, Università degli Studi di Catania, 95124 Catania, Italyspenamariateresa@gmail.com (M.T.S.)

**Keywords:** bats, antimicrobial resistance, wildlife

## Abstract

Bats (order Chiroptera) are increasingly recognized as important reservoirs and potential vectors of pathogenic and antimicrobial-resistant bacteria (ARB), with potential implications for human, animal, and environmental health. This study aimed to assess the presence and antimicrobial resistance profiles of bacterial isolates from bat populations in Sicily, an area for which data are currently limited. A total of 132 samples (120 rectal swabs and 12 guano samples) were collected at four sites in the provinces of Catania, Siracusa, and Ragusa. Bacteriological analysis yielded 213 isolates, including 161 Gram-negative and 52 Gram-positive strains, representing 55 different species. Among Gram-negative isolates, *Escherichia coli*, *Citrobacter freundii*, and *Morganella morganii* were most frequently detected, while *Bacillus licheniformis* and *Staphylococcus xylosus* were predominant among Gram-positive bacteria. Antimicrobial susceptibility testing revealed high resistance rates to colistin, amoxicillin, and ampicillin in Gram-negative strains, and to oxacillin, ceftazidime, and lincomycin in Gram-positive strains. Notably, 84.5% of isolates exhibited multidrug resistance. These findings highlight the potential role of bats as reservoirs of ARB and underline the importance of ongoing monitoring within a One Health framework to mitigate risks to public and animal health.

## Simple Summary

This study investigated the presence of pathogenic bacteria and antibiotic-resistant bacteria in bat populations from southern Italy. Rectal swabs and guano samples were collected from four natural sites located in the provinces of Catania, Siracusa, and Ragusa, in Sicily. A total of 213 bacterial isolates, predominantly Gram-negative species such as *Escherichia coli*, *Citrobacter freundii*, and *Morganella morganii*, were obtained from 132 samples. Antibiotic susceptibility testing revealed high levels of resistance to multiple antibiotics, particularly colistin, amoxicillin, and ampicillin. Notably, 84.5% of the isolates were classified as multidrug-resistant. These findings suggest that Sicilian bats may serve as significant reservoirs and potential vectors of antibiotic-resistant bacteria, highlighting the need for further research to assess the implications for public health and biodiversity conservation.

## 1. Introduction

Bats, belonging to the order *Chiroptera*, represent the second-largest group of mammals, with over 1690 species distributed worldwide [1]. In addition to their broad geographic range, they exhibit unique characteristics such as long lifespans, the ability to fly long distances, adaptability to diverse ecological niches, and, for many species, a strong tendency toward colonial living [2]. These animals play a vital role in global ecosystems and are also recognized as natural reservoirs and carriers of various microorganisms, including viruses, bacteria, fungi, and parasites, some of which are pathogens shared with humans and domestic animals [3]. Recent studies have shifted from focusing exclusively on the presence of pathogens such as *Salmonella*, *Shigella*, *Yersinia*, and *Campylobacter* to a more detailed analysis of the bat microbiota. Bats rely on their gut microbiota to perform vital metabolic functions that are closely linked to their dietary habits. Their microbial composition varies according to their feeding strategies (insectivory, frugivory, nectarivory, carnivory, and hematophagy), and it adjusts dynamically with seasonal dietary changes to meet their nutritional needs [4]. These changes make bats a fascinating subject of study, especially considering their role in the spread of antimicrobial resistance (AMR).

Antimicrobial-resistant bacteria (ARB) have been detected in wild animals that have never been directly exposed to antibiotics [5,6,7]. These bacteria have also been found in environmental samples, suggesting a link to the widespread use of antibiotics, which degrade in natural ecosystems, providing non-lethal doses that favor bacterial survival and adaptability [8,9]. Bats, in particular, are often exposed to diverse microorganisms due to their movement between natural and anthropized environments, which increases their chances of acquiring and potentially spreading a broad range of microbes, including ARBs [10]. The mobility and ecological diversity of bats also contribute to the persistence and transmission of ARBs, not only in anthropized areas but also in agroecosystems and natural ecosystems. Bacteria carried by bats can acquire resistance through interactions with humans and domestic animals, while bats can also transmit resistant bacteria to other species [10,11]. These direct and indirect exchanges play a significant role in the global spread of antimicrobial resistance.

About 60% of the over 1400 bat species occur on islands, and about 25% of all species are island endemics, so they are particularly vulnerable to habitat disturbance caused by urbanization, forest degradation, and cave disturbance [12,13]. The first studies on bats in Sicily, the largest island and the southernmost region of Italy, date back to the 19th century; the distribution and diversity of species on the island have been systematically explored since 1978 [14,15,16,17,18]. Comprehensive accounts of specific cave studies are provided by Massaad et al., 2023 [2]. To date, 28 species have been identified, with the most studied being *Myotis myotis*, *Miniopterus schreibersii*, *Rhinolophus ferrumequinum*, *Myotis capaccinii*, and *Rhinolophus euryale* [1]. The studies conducted on these populations have mainly focused on the taxonomy, ecology, and conservation of these animals. Meanwhile, there are numerically fewer studies investigating health aspects [19,20], such as their possible role as reservoirs and carriers of pathogens, including ARB [7,21].

Despite their ecological importance and their potential role as reservoirs of microorganisms, including antimicrobial-resistant bacteria, the health aspects of Sicilian bat populations remain largely understudied. Given the increasing global concern over antimicrobial resistance and its impact on public health and biodiversity, it is essential to investigate the presence and distribution of ARB in wild animal populations, particularly in species that inhabit natural and anthropized environments.

The aim of this study is to assess the occurrence of pathogenic bacteria and ARB in some populations of troglophilic bats from Eastern Sicily (Italy), analyzing their potential role as reservoirs and vectors of these microorganisms. Through microbiological analyses, we seek to characterize the diversity of ARB within bat populations. These findings will contribute to a better understanding of the role of bats in the ecology of antimicrobial resistance, with implications for wildlife conservation and public health strategies.

## 2. Materials and Methods

This study is part of a broader research project involving a survey on the presence of emerging zoonotic pathogens in bats approved by the Italian Ministry of Health.

### 2.1. Sampling

Samples were collected between 2021 and 2022 from four natural sites located in the provinces of Catania, Siracusa, and Ragusa in Eastern Sicily, Italy. The sampling sites, previously described and mapped in detail [19,20], included Grotta Chiusazza (Floridia, Siracusa), Grotta Caprara (Noto, Siracusa), Grotta del Burrò (Randazzo, Catania), and the Castelluccio Mine (Modica, Ragusa).

Grotta Chiusazza (Lon 15.159536; Lat 37.026406), located at an elevation of 107 m above sea level near Floridia (Siracusa), is a karstic cave with two entrances, one to the northwest and one to the southeast. The cave extends 250 m and has a 15 m negative elevation. The surrounding area consists of farmland characterized by the presence of intensive monocultures and small ponds, where bats often feed and drink. This cave hosts a large colony of *Miniopterus schreibersii* and several Rhinolophids. Grotta Caprara (Lon 14.926690; Lat 37.007520), in the territory of Noto (Siracusa), is part of a series of openings along a rock formation about 200 m above the valley floor. The cave has a wide entrance and extends horizontally for about 100 m with minimal elevation changes. Several hundred *M. schreibersii* and Rhinolophidae call this cave home. The surrounding areas are used for cattle grazing.

Grotta del Burrò (Lon 14.934538; Lat 37.826880), found in Randazzo (Catania), is a 250-m-long lava tube formed within prehistoric lava flows of uncertain age (15 Ka − 3930 ± 60 a). The cave has a 47 m elevation difference, with an entrance located in a funnel-shaped depression. The surrounding shrub–herbaceus vegetation is dense, and semi-wild cattle are raised in the area. A colony of approximately 600–700 *M. schreibersii* bats inhabit the cave, along with larger populations of *Myotis* and bats of the family *Rhinolophidae*.

Miniera di Castelluccio (Lon 14.691320; Lat 36.837110), located near Modica (Ragusa), was once an open-pit mining area for bituminous limestone until the mid-20th century. The site is situated on terraces overlooking the Irminio River. The quarry is nearly flat, measuring approximately 300 m in length and 150 m in width. Around 200 Rhinolophidae (*Rhinolophus ferrumequinum*, *R. euryale*, and *R. hipposideros*) inhabit the site, but no other bat species have been confirmed.

Samples collected from these sites included guano, gathered from the floors of the caves, as well as rectal swabs taken from the bats. The capture of bats was carried out by experienced and authorized personnel (RG and MTS), following the regulations set by the Istituto Superiore per la Protezione e la Ricerca Ambientale (ISPRA) under Authorisation Prot. N. 14589/T-A31(2018) and ISPRA prot. 41518 (approval date: 2 September 2021). Whenever possible, bats were captured by hand to reduce the impact on the colony. In certain caves, a hand-held net with a telescoping handle was employed to capture bats that were stationary or not flying.

After capture, each bat was briefly placed inside a numbered canvas bag, which was closed securely with a cord. Only one bat was placed in each bag. All procedures were in accordance with the guidelines established by the National Institute for Wildlife [22].

Rectal swabs were collected from each bat using individually wrapped sterile microbiological swabs pre-moistened with 0.9% sterile saline solution. The swab tips were gently inserted and rotated against the mucosal surface to obtain samples. Each swab was then placed into a tube containing Amies transport medium (Copan Italia, Brescia, Italy) and kept in an insulated container with frozen gel packs during transport. All bats were handled minimally and released promptly after sampling to reduce stress.

Guano samples were collected from various caves by placing sterile collection plates on the cave floor directly beneath roosting bat colonies. The plates were left in place for approximately two hours to allow for natural guano deposition. Following this period, individual guano pellets were carefully retrieved and immediately stored at 4 °C for transport to the laboratory for subsequent analysis.

All samples were transported on the same day of collection to the Infectious Diseases Laboratory of the Dipartimento di Scienze Veterinarie at the Università degli Studi di Messina, where they were promptly placed in an incubator at 37 °C overnight in tubes containing nutrient broth (Oxoid, Milan, Italy). The following day, the samples were inoculated onto suitable selective media for the isolation of both Gram-negative and Gram-positive bacteria.

### 2.2. Bacterial Isolation and Identification

After enrichment in nutrient broth, the samples were seeded onto selective media to facilitate the isolation of Gram-positive and Gram-negative microorganisms. Specifically, the samples were plated on MacConkey Agar (Biolife Italiana, Milano, Italy), suitable for the growth of Gram-negative bacteria, and on Mannitol Salt agar (Biolife Italiana, Milano, Italy) for Gram-positive isolates. The plates were incubated at 37 °C for 24 h, after which colonies with distinctive macroscopic characteristics were isolated and subsequently transferred to nutrient agar tubes for preservation and further analysis.

The obtained isolates were subcultured on Blood Agar (Biolife Italiana, Milano, Italy) and subsequently identified using MALDI-TOF mass spectrometry (Matrix-Assisted Laser Desorption/Ionization–Time of Flight Mass Spectrometry). The selected colonies were inoculated onto a 48-well metal plate using *Escherichia coli* ATCC 8739 as a control strain. Spectrum analysis was performed using the VITEK MS system (bioMérieux SA, Marcy l’Etoile, France) and Axima Assurance system (Shimadzu, Kyoto, Japan), supported by the SARAMIS database (Spectral Archive and Microbial Identification System, AnagnosTec, Berlin, Germany).

Twenty isolates that could not be identified using MALDI-TOF mass spectrometry were characterized phenotypically using traditional macrotube-based methods following the procedures described by Carter and in Bergey’s manual [23,24]. Bacterial identification was performed through biochemical tests assessing carbohydrate metabolism, motility, enzymatic activities, oxidation–fermentation reactions, and specific metabolic pathways. Finally, serological typing of *Salmonella* spp. isolates was performed according to the Kauffmann–White–Le Minor scheme, in agreement with the National Reference Centre/WOAH Reference Laboratory for Salmonellosis at the Istituto Zooprofilattico Sperimentale delle Venezie (IZSVe), Padua, Italy [25]. The collected isolates were stored at −20 °C pending further analysis.

### 2.3. Antimicrobial Susceptibility Tests

The isolates were then subjected to antibiotic susceptibility testing using the Kirby–Bauer method (disk diffusion method). A bacterial suspension with a density equivalent to 0.5 McFarland standard was first prepared and then streaked onto Mueller–Hinton agar (Biolife Italiana, Milano, Italy) using a sterile swab. Absorbent paper disks impregnated with antimicrobials (Liofilchem S.r.l., Roseto degli Abruzzi, Italy) were placed on the agar surface before incubating the plates at 37 °C for 24 h. Two panels of antimicrobials, one targeting Gram-negative bacteria and the other one targeting Gram-positive bacteria, were tested (Table 1).

After incubation, the diameters of the inhibition zones were measured, and the tested isolates were classified as susceptible, intermediate, or resistant according to the interpretation tables provided by CLSI guidelines [26].

Multidrug-resistant (MDR) bacteria were defined according to an acquired non-susceptibility to at least one agent in three or more antimicrobial categories, as proposed by Magiorakos et al. [27].

## 3. Results

### 3.1. Sampling

During the sampling process, 120 rectal swabs were collected from an equal number of individuals belonging to five species of insectivorous troglophilic bats. These included *Myotis myotis* (*n* = 2), *Miniopterus schreibersii* (*n* = 68), *Rhinolophus euryale* (*n* = 6), *Rhinolophus ferrumequinum* (*n* = 31), and *Rhinolophus hipposideros* (*n* = 13). Additionally, 12 guano samples were collected; 3 in Grotta Burrò and 9 in Grotta Chiusazza. Guano samples were not collected from the other caves due to the absence of suitable environmental conditions for sample collection, such as the insufficient accumulation of fresh guano or limited accessibility beneath roosting sites.

### 3.2. Bacterial Strain Collection

The bacteriological analyses conducted on the 132 samples led to the isolation of 213 bacterial isolates, of which 192 were obtained from rectal swabs and the remaining from guano samples. Gram-negative species were the most frequently isolated. In 33 out of 132 samples analyzed (25%), the coexistence of Gram-negative and Gram-positive strains was detected. A total of 149 Gram-negative and 43 Gram-positive strains were isolated from rectal swabs, while 12 Gram-negative and 9 Gram-positive strains were obtained from guano (Supplementary Table S1). Gram-negative species were the most abundant at all sites, and among these, *Escherichia coli* was the most common species (14.3%), followed by *Citrobacter freundii* (9.9%) and *Morganella morganii* (9.3%). Among the Gram-positive, *Bacillus licheniformis* (33.3%) and *Staphylococcus xylosus* (11.8%) were the most frequently identified species.

**Figure 1:**
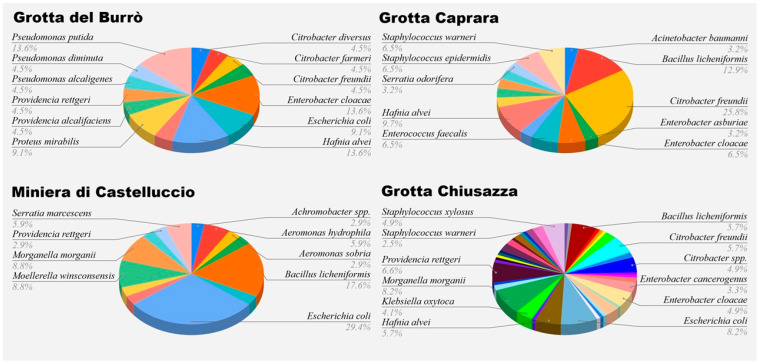
Species identified per sampling site.

As shown in Figure 1, a total of 123 isolates representing 43 different species were collected from Grotta Chiusazza, comprising 25 Gram-negative and 18 Gram-positive species. In Miniera di Castelluccio, 34 isolates were obtained, identifying 14 species, with a notable predominance of Gram-negative species (*n* = 11) over Gram-positive ones (*n* = 3). From Grotta del Burrò, 25 isolates yielded 16 identified species, of which only one was Gram-positive. Lastly, in Grotta Caprara, 31 isolates resulted in the identification of 15 species, including 11 Gram-negative and 3 Gram-positive.

The pie charts show the relative abundance of the bacterial taxa identified in the following locations: Grotta del Burrò, Grotta Caprara, Miniera di Castelluccio, and Grotta Chiusazza. Each color segment represents a distinct bacterial species, with the corresponding percentage indicating its proportion of the total number of isolates from that site.

From the 192 isolates obtained from rectal swabs, 55 different bacterial species were identified. A total of 32 species were detected in the fecal samples of *Miniopterus schreibersii*, followed by 26 species in the fecal samples of *Rhinolophus ferrumequinum*, and 25 species in the fecal samples of *Rhinolophus hipposideros*. Additionally, three different species were found in the fecal samples of *Myotis myotis*, and eight species were identified in the fecal samples of *Rhinolophus euryale.* The bacterial species and the number of isolates collected from the different bat species analyzed are presented in Supplementary Table S2.

### 3.3. Antimicrobial Susceptibility Tests

Susceptibility testing was performed on the 149 Gram-negative isolates and the 43 Gram-positive isolates that were collected from rectal swabs. Of the tested isolates, 96.6% (144/149) showed resistance to one or more molecules, while only five isolates (CAP_N1, CH_N7, CH_N24, CH_N25, and CH_N28) showed no resistance apart from resistance known to be intrinsic to the identified species (Table S3).

Most of the Gram-negative isolates, predominantly belonging to the Enterobacteriaceae family, showed resistance to amoxicillin (65.8%), streptomycin (61.1%), and imipenem (43.6%). In contrast, very low resistance percentages were observed for fluoroquinolones (Table 2).

Gram-positive isolates exhibited resistance to oxacillin (74.4%), lincomycin (65.1%), tobramycin (53.5%), and vancomycin (51.2%) (Table S4). In contrast, the lowest resistance rates were observed for ampicillin + sulbactam and minocycline, with only one resistant strain (2.3%) (Table 3).

Of the 149 isolates, 126 were MDR, 87 were Gram-negative, and 39 were Gram-positive (Table S5). Specifically, 40 different MDR profiles were detected for Gram-negatives, and the most prominent MDR profile was resistance to aminoglycosides, penicillins, and tetracyclines, which was detected in 10 isolates: one *Citrobacter* spp. (CH_N14), one *Citrobacter diversus* (B3), one *Enterobacter hormaechei* (CH_N31), one *Escherichia coli* (CH_N41), one *Hafnia alvei* (CAS_N14), one *Klebsiella oxytoca* (CH_N48), three *Providencia rettgeri* (CH_N67, CH_N66, and CH_N72), and one *Morganella morganii* (CAP_N18). Among the Gram-positives, 26 different MDR profiles were detected, and the most frequent were resistance to four classes at the same time, i.e., aminoglycosides, carbapenems, fluoroquinolones, lincosamides, macrolides, and penicillins detected in 4/39 isolates, and resistance to aminoglycosides, cephalosporins, lincosamides, and penicillins detected in a further four isolates.

## 4. Discussion

This preliminary study examined the diversity and antimicrobial resistance of cultivable gastrointestinal bacteria isolated from bat fecal and guano samples collected in four caves in Eastern Sicily. The bacteriological analyses provided valuable insights into the composition of the gastrointestinal microbiota of insectivorous bats and assessed the presence of potentially pathogenic bacteria. The microbiota plays a crucial role in host evolution and contributes to several essential physiological functions, including sugar metabolism [28], digestion and nutrient absorption [29,30], and the production of metabolic enzymes [31].

In this study, we identified 213 bacterial isolates belonging to 62 different species distributed across 25 genera (20 Gram-negative and 5 Gram-positive). Some of the isolated species are considered commensal or environmental contaminants, such as *Bacillus* spp., while others, including *Salmonella enterica* and *Pseudomonas aeruginosa*, are potentially pathogenic to bats, other animals, and humans [3,32,33]. The most frequently isolated genera were *Citrobacter* (14%) and *Enterobacter* (11%). Although the bacterial genera identified were present in all the examined populations, the specific species varied depending on the site. However, *Hafnia alvei* was the only species identified in all the analyzed populations. These results are consistent with previous studies conducted on bats in Eastern Sicily, further supporting the idea of a shared microbial composition among bat populations likely due to dietary and environmental factors [21,34]. The presence of *Proteobacteria* and *Firmicutes* phyla as dominant groups in bat guano or fecal samples is consistent with other previous studies [35,36,37]. Although many genera in these studies were also found in our research, variations in isolated species may result from different feeding habits. This suggests that diet plays a crucial role in the formation of the gut microbiota, contributing to the microbial similarities between different bat species. Bacteria found in insects, the main food source for these bats, can colonize their gastrointestinal tract. Indeed, many microorganisms previously isolated from insects have been found in the microbiota of insectivorous bats [21,36,38]. These results emphasize the importance of investigating bat microbiomes from different geographic regions with comparable habitats to assess whether a symbiotic microbial core has evolved alongside its host.

We observed significant levels of resistance to common antimicrobials, reflecting trends seen in other wildlife studies. In particular, resistance to amoxicillin (65.8%) was common among Gram-negative bacteria, a worrying finding as this agent is typically used to treat infections caused by *Enterobacteriaceae* and other pathogens. The high resistance to these antibiotics in wildlife populations like bats is concerning because it could limit the effectiveness of these drugs when treating human and animal infections. This resistance is likely to reflect widespread environmental exposure to antimicrobials, as seen in other European bat studies [7,39,40,41,42]. These findings suggest that antimicrobial resistance in bats may result from both environmental exposure and the ingestion of insects carrying resistant strains [42,43]. As natural bacterial vectors, flying insects can spread resistant bacteria across different ecosystems. When consumed by insectivorous bats, these bacteria may persist in their gut microbiota and contribute to environmental contamination. Moreover, resistant strains found in insects often share genetic similarities with those detected in humans and animals living in overlapping habitats, highlighting the role of insect prey in the spread of antimicrobial resistance [36,39,44]. This indicates that insect-borne AMR can act as a bridge for the transmission of ARB between wildlife, domestic animals, and humans. This close interaction between bats and their insect prey creates a potential route for the transmission of resistant bacteria within bat populations.

The high level of MDR among bat isolates and their resistance profiles suggests that bats serve as long-term reservoirs for ARB. Their relatively long lifespans (*Miniopterus schreibersii* has an average age 2–3 years, with a maximum recorded age of 16 years; *Myotis myotis* has a maximum longevity of 22 years, with an average lifespan of 4–5 years; *Rhinolophus ferrumequinum* has a maximum recorded age of 30 years; and *Rhinolophus hipposideros* has a maximum recorded age of 21 years) allow them to harbor resistant strains for extended periods, increasing the likelihood of persistence within bat populations and potential transmission to other species through direct contact or environmental exposure.

As shown in Table S5, some isolates obtained from individuals belonging to different bat species cohabiting in the same cave displayed overlapping MDR profiles. This pattern was observed in Grotta Caprara, Grotta Chiusazza, and Miniera di Castelluccio, where isolates from different bacterial taxa and from different bat species exhibited similar resistance patterns. These findings suggest that physical proximity and shared environments may facilitate the circulation or exchange of ARB among sympatric bat species. Conversely, individuals of the same bat species sampled in different caves often harbored bacteria with distinct resistance profiles, indicating that resistance traits may be shaped more by cave-specific ecological conditions than by host species alone. These observations point to both convergence and divergence in resistance profiles depending on host and environmental contexts. Although these results highlight patterns of similarity and divergence in MDR profiles, they do not allow us to conclusively attribute the observed variations to specific ecological or behavioral factors. However, it is plausible that factors such as roosting behavior, social structure, interspecies contact within roosts, and differences in foraging strategies or habitat use may influence the acquisition and dissemination of resistant bacteria. Further research is required to determine whether sympatric bats share resistant microbiota primarily through direct contact or environmental exposure and whether allopatric populations of the same species diverge in their resistance profiles due to distinct ecological pressures in different habitats. Each cave under study hosts bat colonies, defined by species and population size [19]. Although the caves are located approximately 30 to 100 km apart, no migration has been observed between bat populations inhabiting different caves. However, periodic migration of bats from the caves occurs, although the destination of these movements remains unknown, with the bats eventually returning.

Bat behavior, particularly colonial living, further facilitates the spread of antimicrobial resistance. Close contact within bat colonies enhances bacterial transmission, reinforcing the persistence of resistant strains. Additionally, environmental contamination from bat feces, which often contains resistant bacteria, contributes to the dissemination of antimicrobial resistance in their habitats, potentially affecting other wildlife, livestock, and humans in close proximity who frequent their foraging areas.

Our findings highlight the important role of bats in the persistence and spread of antimicrobial resistance, aligning with studies across Europe that increasingly recognize wildlife as significant reservoirs of resistant bacteria. This underlines the importance of including wild animals in AMR strategies for surveillance and control, following a One Health approach.

The absence of molecular analyses represents a significant limitation of this study. Such analyses would have provided valuable insights into the genetic mechanisms driving antimicrobial resistance and facilitated comparative assessments with isolates from humans and livestock, thereby enhancing the understanding of potential epidemiological links. Future studies incorporating targeted molecular approaches will be crucial to address this limitation and deepen our knowledge of resistance dynamics.

## 5. Conclusions

This study provides an assessment of the gastrointestinal microbiota and AMR of some troglophilus bat populations in Sicily. The results indicate a complex microbial composition, with commensal and potentially pathogenic bacteria present in the gastrointestinal tract of these bats. The levels of antimicrobial resistance observed raise concerns about the possibility of bats acting as reservoirs of resistant bacterial strains. These resistant bacteria can be acquired by bats through environmental exposure and ingestion of prey insects, which then act as vectors contributing to the continued spread of resistance in ecosystems, with implications for human and animal health.

In conclusion, the results of this study emphasize the need for continuous surveillance of AMR in wildlife to better understand the dynamics of resistance in natural ecosystems.

## Figures and Tables

**Table 1:** Antimicrobials used in this study.

	Antimicrobials Classes	Molecules
Gram-negative Panel	Aminoglycosides	Gentamicin
		Streptomycin
		Tobramycin
	Carbapenems	Imipenem
		Meropenem
	Cephalosporins	Cefotaxime
		Ceftazidime
		Ceftazidime + clavulanic acid
	Quinolones	Nalidixic acid
	Chloramphenicol	Chloramphenicol
	Fluoroquinolones	Ciprofloxacin
		Enrofloxacin
	Monobactams	Aztreonam
	Penicillins	Amoxicillin
		Amoxicillin + clavulanic acid
		Ampicillin
	Sulphonamides	Cotrimoxazole
	Tetracyclines	Tetracycline
		Doxycyclin
Gram-positive Panel	Aminoglycosides	Gentamicin
		Tobramycin
	Carbapenems	Imipenem
		Meropenem
	Cephalosporins	Ceftazidime
		Cefepime
		Ceftaroline
	Fluoroquinolones	Enrofloxacin
	Glycopeptides	Vancomycin
	Lincosamides	Lincomycin
	Macrolides	Erythromycin
	Penicillins	Amoxicillin
		Ampicillin
		Oxacillin
		Amoxicillin + clavulanic acid
		Ampicillin + sulbactam
		Ticarcillin + clavulanic acid
	Tetracyclines	Minocycline
		Tetracycline

**Table 2:** Gram-negative isolates’ antimicrobial susceptibility patterns.

Classes	Molecules	Number of Isolates (%)		
		R	I	S
Aminoglycosides	Gentamicin	51 (34.2)	1 (0.7)	97 (65.1)
	Streptomycin	91 (61.1)	6 (4)	52 (34.9)
	Tobramycin	57 (38.3)	3 (2)	89 (59.7)
Carbapenems	Imipenem	65 (43.6)	5 (3.4)	79 (53)
	Meropenem	9 (6)	3 (2)	137 (91.9)
Cephalosporins	Cefotaxime	34 (22.8)	5 (3.4)	110 (73.8)
	Ceftazidime	26 (17.4)	3 (2)	120 (80.5)
	Ceftazidime + clavulanic acid	16 (10.7)	6 (4)	127 (85.2)
Quinolones	Nalidixic acid	18 (12.1)	9 (6)	122 (81.9)
Chloramphenicol	Chloramphenicol	12 (8.1)	3 (29)	134 (89.9)
Fluoroquinolones	Ciprofloxacin	6 (4)	0	143 (96)
	Enrofloxacin	0	2 (1.3)	147 (98.7)
Monobactams	Aztreonam	21 (14.1)	1 (0.7)	127 (85.2)
Penicillins	Amoxicillin	98 (65.8)	1 (0.7)	50 (33.6)
	Amoxicillin + clavulanic acid	44 (29.5)	4 (2.7)	101(67.8)
	Ampicillin	38 (25.5)	3 (2)	108 (72.5)
Sulphonamides	Trimethoprim-Sulfamethoxazole	10 (6.7)	1 (0.7)	138 (92.6)
Tetracyclines	Tetracycline	38 (25.5)	2 (1.3)	109 (73.2)
	Doxycycline	44 (29.5)	11 (7.4)	94 (63.1)

**Table 3:** Gram-positive strain antimicrobial susceptibility patterns.

Classes	Molecules	Number of Isolates (%)		
		R	I	S
Aminoglycosides	Gentamicin	20 (46.5)	0	23 (53.5)
	Tobramycin	23 (53.5)	0	20 (46.5)
Carbapenems	Imipenem	14 (32.6)	3 (7)	26 (60.5)
	Meropenem	11 (25.6)	2 (4.7)	30 69.8)
Cephalosporins	Ceftazidime	14 (32.6)	0	29 (67.4)
	Cefepime	14 (32.6)	1 (2.3)	28 (65.1)
	Ceftaroline	14 (32.6)	1 (2.3)	28 (65.1)
Fluoroquinolones	Enrofloxacin	8 (18.6)	0	35 (81.4)
Glycopeptides	Vancomycin	22 (51.2)	1 (2.3)	20 (46.5)
Lincosamides	Lincomycin	31 (72.1)	2 (4.7)	10 (23.3)
Macrolides	Erythromycin	19 (44.2)	3 (7)	21 (48.8)
Penicillins	Amoxicillin	16 (37.2)	0	27 (62.8)
	Ampicillin	21 (48.8)	0	22 (51.2)
	Oxacillin	32 (74.4)	0	11 (25.6)
	Amoxicillin + clavulanic acid	12 (27.9)	0	31 (72.1)
	Ampicillin + sulbactam	1 (2.3)	0	42 (97.7)
	Ticarcillin + clavulanic acid	7 (16.3)	1 (2.3)	35 (81.4)
Tetracyclines	Minocycline	1 (2.3)	2 (4.7)	40 (93)
	Tetracycline	2 (4.7)	0	41 (95.3)

## Data Availability

Data are contained within the article.

## Supplementary Materials

The following supporting information can be downloaded at: https://www.mdpi.com/article/10.3390/vetsci12060516/s1, Table S1: Isolation and identification results. Table S2: Bacterial species isolated from the bat species under investigation. Table S3: Gram-negative samples from bats: bacterial species identification, bat species, sampling site, and susceptibility data. Table S4: Gram-positive samples from bats: bacterial species identification, bat species, sampling site, and susceptibility data. Table S5: MDR patterns of bacterial isolates from bats: host species and cave of origin.

## Abbreviations

The following abbreviations are used in this manuscript:

AMR	Antimicrobial resistance.
MDR	Multidrug-resistant.
ARB	Antimicrobial-resistant bacteria.
