# Supplementary material for: Exploring Antimicrobial Resistance in Bacteria from Fecal Samples of Insectivorous Bats: A Preliminary Study
Source: Vet Sci. 2025 May 25;12(6):516. doi: 10.3390/vetsci12060516 (PMC12197713; doi:10.3390/vetsci12060516)
Supplement: Supplementary file 1 [file vetsci-12-00516-s001.zip › vetsci-3610869-supplementary.pdf]

Table S1. Isolation and identification results.

Sample	*Bacterial Family*	*Bacterial Species*	*No. of Isolates*
Rectal swab	Alcaligenaceae	*Alcaligenes faecalis*	2
	Bacillaceae	*Bacillus atrophaeus*	1
		Bacillus cereus	1
		Bacillus licheniformis	13
		Bacillus safensis	1
		Bacillus subtilis	1
		Bacillus velezensis	1
		Cytobacillus horneckiae	1
	Enterobacteriaceae	*Achromobacter* spp.	1
		Acinetobacter baumanni	1
		Aeromonas hydrophila	2
		Aeromonas sobria	1
		Citrobacter braakii	3
		Citrobacter diversus	1
		Citrobacter farmeri	1
		Citrobacter freundii	15
		Citrobacter gillenii	2
		Citrobacter spp.	6
		Citrobacter werkmanii	1
		Enterobacter asburiae	3
		Enterobacter cancerogenus	5
		Enterobacter cloacae	10
		Enterobacter farmeri	1
		Enterobacter hormaechei	4
		Enterobacter spp.	1
		Escherichia coli	22
		Flavobacterium breve	1
		Hafnia alvei	9
		Klebsiella oxytoca	5
		Moellerella winsconsensis	3
		Morganella morganii	15
		Proteus mirabilis	4
		Proteus vulgaris	1
		Providencia rettgeri	10
		Pseudocitrobacter faecalis	1
		Rahnella aquatilis	1
		Salmonella enterica subsp. Houtenae	1
		Salmonella enterica subsp. Arizonae	1
		Serratia liquefaciens	1
		Serratia marcescens	6
		Serratia odorifera	1
		Serratia spp.	2
	Enterococcaceae	*Enterococcus casselliflavus*	1
		Enterococcus faecalis	3
	Pasteurellaceae	*Pasteurella aerogenes*	1

	Pseudomonadaceae	*Pseudomonas aeruginosa*	1
		Pseudomonas alcaligenes	1
		Pseudomonas putida	2
	Staphylococcaceae	*Staphylococcus capitis*	1
		Staphylococcus cohnii	2
		Staphylococcus epidermidis	3
		Staphylococcus sciuri	2
		Staphylococcus simulans	1
		Staphylococcus warneri	5
		Staphylococcus xylosus	6
Guano	Enterobacteriaceae	*Citrobacter freundii*	1
		Enterobacter cloacae	1
		Escherichia coli	1
		Hafnia alvei	5
		Providencia alcalifaciens	1
		Serratia fonticola	1
	Bacillaceae	*Bacillus amiloliquefaciens*	*1*
		Bacillus circulans	*1*
		Bacillus licheniformis	*4*
		Bacillus pumilus	*1*
		Lysinibacillus fusiformis	*1*
	Enterococcaceae	*Enterococcus faecium*	*1*
	Pseudomonadaceae	*Pseudomonas diminuta*	1
		Pseudomonas putida	1

Table S2. Bacterial species isolated from the bat species under investigation.

Miniopterus schreibersii		*Rhinolophus ferrumequinum*		*Rhinolophus hipposideros*		*Rhinolophus euryale*		*Myotis myotis*	
**Bacterial Species**	**No.**	**Bacterial Species**	**No.**	**Bacterial Species**	**No.**	**Bacterial Species**	**No.**	**Bacterial Species**	**No.**
Alcaligenes faecalis	1	*Achromobacter spp.*	1	*Bacillus licheniformis*	2	*Citrobacter braakii*	1	*Alcaligenes faecalis*	1
Bacillus cereus	1	*Acinetobacter baumanni*	1	*Citrobacter freundii*	1	*Citrobacter freundii*	1	*Citrobacter diversus*	1
Bacillus licheniformis	5	*Aeromonas hydrophila*	2	*Citrobacter spp.*	1	*Citrobacter spp.*	1	*Hafnia alvei*	1
Bacillus safensis	1	*Aeromonas sobria*	1	*Citrobacter werkmanii*	1	*Enterobacter cloacae*	1		
Bacillus velezensis	1	*Bacillus atrophaeus*	1	*Enterobacter cancerogenus*	4	*Enterobacter hormaechei*	2		
Citrobacter braakii	2	*Bacillus licheniformis*	6	*Enterobacter cloacae*	6	*Escherichia coli*	1		
Citrobacter farmeri	1	*Bacillus subtilis*	1	*Enterobacter hormaechei*	2	*Hafnia alvei*	2		
Citrobacter freundii	8	*Citrobacter freundii*	5	*Enterobacter spp.*	1	*Staphylococcus warneri*	1		
Citrobacter spp.	3	*Citrobacter gillenii*	2	*Enterococcus casselliflavus*	1				
Cytobacillus horneckiae	1	*Citrobacter spp.*	1	*Enterococcus faecalis*	1				
Enterobacter asburiae	3	*Enterobacter farmeri*	1	*Escherichia coli*	3				
Enterobacter cancerogenus	1	*Escherichia coli*	12	*Hafnia alvei*	1				
Enterobacter cloacae	3	*Flavobacterium breve*	1	*Klebsiella oxytoca*	4				
Enterococcus faecalis	2	*Hafnia alvei*	1	*Moellerella winsconsensis*	1				
Escherichia coli	6	*Moellerella winsconsensis*	2	*Morganella morganii*	3				
Hafnia alvei	4	*Morganella morganii*	6	*Proteus mirabilis*	2				
Klebsiella oxytoca	1	*Pasteurella aerogenes*	1	*Providencia rettgeri*	4				
Morganella morganii	5	*Proteus vulgaris*	1	*Pseudomonas alcaligenes*	1				
Proteus mirabilis	2	*Providencia rettgeri*	5	*Pseudomonas putida*	1				
Providencia rettgeri	1	*Serratia marcescens*	3	*S. enterica* subsp. Arizonae	1				
Pseudocitrobacter faecalis	1	*Serratia odorifera*	1	*S. enterica* subsp. Houtenae	1				
Pseudomonas aeruginosa	1	*Staphylococcus capitis*	1	*Serratia marcescens*	2				
Pseudomonas putida	1	*Staphylococcus epidermidis*	1	*Staphylococcus cohnii*	1				
Rahnella aquatilis	1	*Staphylococcus sciuri*	1	*Staphylococcus sciuri*	1				
Serratia liquefaciens	1	*Staphylococcus warneri*	2	*Staphylococcus warneri*	1				
Serratia marcescens	1	*Staphylococcus xylosus*	1						
Serratia spp.	2								
Staphylococcus cohnii	1								
Staphylococcus epidermidis	2								
Staphylococcus simulans	1								
Staphylococcus warneri	1								
Staphylococcus xylosus	5								

Table S3. Gram-negative from bats: bacterial species identification, bats species, sampling site, and susceptibility data.

Isolate ID	Bacterial Species	Bats Species	Sampling Site	CN	STR	TOB	IMI	MERO	CTX	CAZ	CPT	NAL	CHL	CIP	ENRO	AZT	AMX	AMC	AMP	SXT	TET	DOX
B1	*Alcaligenes faecalis*	*Miniopterus schreibersii*	G. Burrò	R	R	S	S	S	S	S	S	S	S	S	S	S	R	R	R	S	S	S
B2	*Alcaligenes faecalis*	*Myotis myotis*	G. Burrò	S	R	S	S	S	R	S	I	S	S	S	S	S	R	R	R	S	S	S
B3	*Citrobacter diversus*	*Myotis myotis*	G. Burrò	S	R	R	S	S	S	S	S	S	S	S	S	S	R	R	R	S	R	I
B4	*Citrobacter farmeri*	*Miniopterus schreibersii*	G. Burrò	R	S	S	S	S	S	S	S	S	S	S	S	S	R	R	R	S	S	S
B5	*Citrobacter freundii*	*Rhinolophus euryale*	G. Burrò	R	R	S	I	R	R	S	S	R	S	S	S	S	a	a	S	S	S	R
B6	*Enterobacter cancerogenus*	*Miniopterus schreibersii*	G. Burrò	R	S	S	S	S	S	S	S	S	S	S	S	S	I	S	R	S	S	S
B7	*Enterobacter cloacae*	*Rhinolophus euryale*	G. Burrò	S	R	S	S	S	S	S	S	S	R	S	S	S	a	a	a	R	R	I
B8	*Enterobacter cloacae*	*Miniopterus schreibersii*	G. Burrò	S	R	S	S	S	S	S	S	S	S	S	S	S	a	a	a	S	S	S
B9	*Enterobacter cloacae*	*Miniopterus schreibersii*	G. Burrò	S	R	S	S	S	R	S	I	R	S	S	S	R	a	a	S	S	S	R
B10	*Escherichia coli*	*Rhinolophus ferrumequinum*	G. Burrò	R	R	S	R	S	R	I	S	R	S	R	I	R	R	R	R	S	R	I
B11	*Escherichia coli*	*Rhinolophus euryale*	G. Burrò	R	R	S	R	S	R	S	S	R	S	R	I	R	R	R	S	S	S	S
B12	*Hafnia alvei*	*Miniopterus schreibersii*	G. Burrò	R	R	S	R	S	R	S	S	S	R	R	S	S	S	S	a	S	R	R
B13	*Hafnia alvei*	*Rhinolophus euryale*	G. Burrò	S	S	S	R	S	S	S	S	S	S	S	S	S	R	a	a	S	S	S
B14	*Morganella morganii*	*Myotis myotis*	G. Burrò	S	R	R	R	S	R	R	S	S	S	S	S	R	R	a	a	R	R	R
B15	*Proteus mirabilis*	*Miniopterus schreibersii*	G. Burrò	S	S	I	S	S	S	S	I	S	S	S	S	S	R	R	R	S	S	S
B16	*Proteus vulgaris*	*Rhinolophus ferrumequinum*	G. Burrò	S	R	S	S	S	S	S	S	S	S	S	S	S	S	S	S	S	S	a
B17	*Providencia rettgeri*	*Rhinolophus ferrumequinum*	G. Burrò	S	S	S	R	S	S	S	S	S	R	S	S	S	R	a	a	S	S	a
B18	*Pseudomonas putida*	*Miniopterus schreibersii*	G. Burrò	S	R	S	S	S	S	S	S	S	S	S	S	S	R	R	R	S	S	S
CAP_N1	*Acinetobacter baumanni*	*Rhinolophus euryale*	G. Caprara	I	S	S	S	S	I	S	S	S	a	S	S	a	a	a	a	S	S	S
CAP_N2	*Citrobacter braakii*	*Rhinolophus euryale*	G. Caprara	R	R	R	R	R	R	R	S	S	S	S	S	S	R	R	S	S	S	R
CAP_N3	*Citrobacter freundii*	*Miniopterus schreibersii*	G. Caprara	R	R	S	S	S	S	S	S	S	S	S	S	S	a	a	S	S	S	S
CAP_N4	*Citrobacter freundii*	*Miniopterus schreibersii*	G. Caprara	S	R	S	S	S	S	S	S	S	S	S	S	S	a	a	S	S	S	S
CAP_N5	*Citrobacter freundii*	*Miniopterus schreibersii*	G. Caprara	R	S	R	S	S	S	S	S	S	S	S	S	S	S	S	S	S	S	S
CAP_N6	*Citrobacter freundii*	*Miniopterus schreibersii*	G. Caprara	R	S	R	S	S	S	S	S	S	S	S	S	S	a	a	S	S	S	S
CAP_N7	*Citrobacter freundii*	*Rhinolophus ferrumequinum*	G. Caprara	S	S	S	S	S	S	S	S	S	S	S	S	S	a	a	S	S	S	S
CAP_N8	*Citrobacter freundii*	*Rhinolophus ferrumequinum*	G. Caprara	R	S	R	S	S	S	S	S	S	S	S	S	S	S	S	S	S	S	S

CAP_N9	*Citrobacter freundii*	*Rhinolophus ferrumequinum*	G. Caprara	S	S	S	S	S	S	S	S	S	S	S	S	R	S	S	S	S	S	S
CAP_N10	*Citrobacter spp.*	*Rhinolophus euryale*	G. Caprara	R	R	R	R	S	S	R	R	R	S	S	S	R	R	R	R	S	S	S
CAP_N11	*Enterobacter asburiae*	*Miniopterus schreibersii*	G. Caprara	S	R	R	R	S	S	S	S	S	S	S	S	S	R	a	a	S	S	S
CAP_N12	*Enterobacter cloacae*	*Miniopterus schreibersii*	G. Caprara	S	R	S	S	S	S	S	S	S	R	S	S	S	a	a	a	R	R	R
CAP_N13	*Enterobacter cloacae*	*Miniopterus schreibersii*	G. Caprara	S	S	S	R	S	S	S	S	S	S	S	S	S	a	a	a	S	S	S
CAP_N14	*Enterobacter hormaechei*	*Rhinolophus euryale*	G. Caprara	S	R	R	R	S	S	S	S	S	S	S	S	S	R	a	a	S	S	S
CAP_N15	*Enterobacter hormaechei*	*Rhinolophus euryale*	G. Caprara	R	S	R	S	S	S	S	S	S	S	S	S	S	S	S	a	S	S	S
CAP_N16	*Escherichia coli*	*Miniopterus schreibersii*	G. Caprara	S	R	S	S	S	S	S	S	S	S	S	S	S	R	R	R	R	R	R
CAP_N17	*Hafnia alvei*	*Rhinolophus euryale*	G. Caprara	S	S	S	S	S	S	S	S	S	S	S	S	S	R	a	a	S	S	S
CAP_N18	*Morganella morganii*	*Rhinolophus ferrumequinum*	G. Caprara	S	R	R	S	S	S	S	S	S	S	S	S	S	R	a	a	S	R	R
CAP_N19	*Pseudocitrobacter faecalis*	*Miniopterus schreibersii*	G. Caprara	R	R	R	R	S	R	S	S	S	S	S	S	S	R	R	R	S	R	I
CAP_N20	*Pseudomonas aeruginosa*	*Miniopterus schreibersii*	G. Caprara	R	R	R	I	R	a	S	S	R	a	S	S	S	a	a	a	R	R	R
CAP_N21	*Serratia marcescens*	*Rhinolophus ferrumequinum*	G. Caprara	R	R	R	S	S	S	S	S	S	S	S	S	S	R	a	a	S	R	R
CAP_N22	*Serratia odorifera*	*Rhinolophus ferrumequinum*	G. Caprara	R	R	S	S	S	R	S	S	S	S	S	S	S	S	S	S	S	R	R
CH_N1	*Citrobacter braakii*	*Miniopterus schreibersii*	G. Chiusazza	R	S	S	S	S	S	S	S	S	S	S	S	S	R	R	S	S	S	S
CH_N2	*Citrobacter braakii*	*Miniopterus schreibersii*	G. Chiusazza	S	S	S	S	S	R	S	I	I	S	S	S	S	S	S	R	S	I	S
CH_N3	*Citrobacter freundii*	*Rhinolophus hipposideros*	G. Chiusazza	R	R	R	R	R	S	R	R	S	S	S	S	S	a	a	S	S	S	R
CH_N4	*Citrobacter freundii*	*Miniopterus schreibersii*	G. Chiusazza	S	R	R	S	R	S	R	R	S	S	S	S	S	a	a	S	S	S	R
CH_N5	*Citrobacter freundii*	*Miniopterus schreibersii*	G. Chiusazza	R	R	S	R	R	S	R	R	R	S	S	S	S	S	S	a	S	S	S
CH_N6	*Citrobacter freundii*	*Rhinolophus ferrumequinum*	G. Chiusazza	R	R	S	R	R	R	R	S	S	S	S	S	S	a	a	a	S	S	S
CH_N7	*Citrobacter freundii*	*Rhinolophus ferrumequinum*	G. Chiusazza	S	S	S	I	S	S	S	S	S	S	S	S	S	S	S	a	S	S	S
CH_N8	*Citrobacter freundii*	*Rhinolophus hipposideros*	G. Chiusazza	R	S	R	I	S	S	S	S	S	S	S	S	S	a	a	a	S	S	S
CH_N9	*Citrobacter freundii*	*Miniopterus schreibersii*	G. Chiusazza	S	S	S	R	S	S	S	S	S	S	S	S	S	a	a	S	S	S	S
CH_N10	*Citrobacter gillenii*	*Rhinolophus ferrumequinum*	G. Chiusazza	R	R	S	R	S	S	R	R	R	S	S	S	S	S	S	R	S	S	S
CH_N11	*Citrobacter gillenii*	*Rhinolophus ferrumequinum*	G. Chiusazza	S	R	S	R	S	S	R	R	R	S	S	S	S	S	S	R	S	S	S
CH_N12	*Citrobacter spp.*	*Miniopterus schreibersii*	G. Chiusazza	S	R	R	R	S	S	R	R	R	S	S	S	R	R	R	R	S	S	S
CH_N13	*Citrobacter spp.*	*Miniopterus schreibersii*	G. Chiusazza	S	R	S	R	S	S	R	S	S	S	S	S	S	R	R	S	S	R	I
CH_N14	*Citrobacter spp.*	*Miniopterus schreibersii*	G. Chiusazza	S	R	R	S	S	S	S	S	S	S	S	S	S	R	R	S	S	R	R
CH_N15	*Citrobacter spp.*	*Rhinolophus ferrumequinum*	G. Chiusazza	S	S	S	S	S	S	S	S	S	S	S	S	S	R	R	R	S	S	S
CH_N16	*Citrobacter spp.*	*Rhinolophus hipposideros*	G. Chiusazza	R	S	S	S	S	S	S	S	I	S	S	S	S	S	S	R	S	S	S

CH_N17	*Citrobacter werkmanii*	*Rhinolophus hipposideros*	G. Chiusazza	S	S	S	S	S	S	S	S	I	S	S	S	S	R	R	R	S	S	S
CH_N18	*Enterobacter asburiae*	*Miniopterus schreibersii*	G. Chiusazza	R	R	S	R	S	R	S	I	R	S	S	S	R	R	a	a	R	R	I
CH_N19	*Enterobacter asburiae*	*Miniopterus schreibersii*	G. Chiusazza	S	R	S	R	S	S	S	S	R	S	S	S	R	R	a	a	S	S	R
CH_N20	*Enterobacter cancerogenus*	*Rhinolophus hipposideros*	G. Chiusazza	S	R	S	S	S	S	S	S	S	R	S	S	S	R	R	R	R	R	I
CH_N21	*Enterobacter cancerogenus*	*Rhinolophus hipposideros*	G. Chiusazza	S	S	S	S	S	S	S	S	S	S	S	S	S	R	R	R	S	S	S
CH_N22	*Enterobacter cancerogenus*	*Rhinolophus hipposideros*	G. Chiusazza	S	S	S	S	S	S	S	S	S	S	S	S	S	R	I	I	S	S	S
CH_N23	*Enterobacter cancerogenus*	*Rhinolophus hipposideros*	G. Chiusazza	R	S	S	S	S	S	S	S	S	S	S	S	S	R	R	I	S	S	S
CH_N24	*Enterobacter cloacae*	*Rhinolophus hipposideros*	G. Chiusazza	S	S	S	S	S	S	S	S	I	S	S	S	S	a	a	a	S	S	S
CH_N25	*Enterobacter cloacae*	*Rhinolophus hipposideros*	G. Chiusazza	S	S	S	S	S	S	S	S	S	S	S	S	S	a	a	a	S	S	S
CH_N26	*Enterobacter cloacae*	*Rhinolophus hipposideros*	G. Chiusazza	R	S	S	S	S	S	S	S	S	S	S	S	S	a	a	a	S	S	S
CH_N27	*Enterobacter cloacae*	*Rhinolophus hipposideros*	G. Chiusazza	S	R	R	R	S	S	S	S	S	S	S	S	S	a	a	a	S	S	S
CH_N28	*Enterobacter cloacae*	*Rhinolophus hipposideros*	G. Chiusazza	S	S	S	S	S	S	S	S	I	S	S	S	S	S	S	a	S	S	S
CH_N29	*Enterobacter farmeri*	*Rhinolophus ferrumequinum*	G. Chiusazza	S	R	S	S	S	S	S	S	S	S	S	S	S	R	R	S	S	S	S
CH_N30	*Enterobacter hormaechei*	*Rhinolophus hipposideros*	G. Chiusazza	R	R	R	S	S	R	S	S	S	S	S	S	S	S	S	a	S	R	I
CH_N31	*Enterobacter hormaechei*	*Rhinolophus hipposideros*	G. Chiusazza	S	R	R	S	S	S	S	S	S	S	S	S	S	R	a	S	S	R	R
CH_N32	*Enterobacter spp.*	*Rhinolophus hipposideros*	G. Chiusazza	R	R	S	S	S	S	S	S	S	S	S	S	S	R	R	S	S	S	S
CH_N33	*Escherichia coli*	*Rhinolophus hipposideros*	G. Chiusazza	R	R	S	R	S	R	S	S	R	S	S	S	R	R	R	S	S	R	R
CH_N34	*Escherichia coli*	*Rhinolophus hipposideros*	G. Chiusazza	R	S	S	R	S	R	S	S	S	S	S	S	R	R	R	S	S	S	R
CH_N35	*Escherichia coli*	*Rhinolophus hipposideros*	G. Chiusazza	S	R	S	R	S	S	R	R	S	S	S	S	S	R	R	S	S	R	R
CH_N36	*Escherichia coli*	*Rhinolophus ferrumequinum*	G. Chiusazza	S	R	R	R	S	S	R	R	S	S	S	S	S	R	R	R	S	S	R
CH_N37	*Escherichia coli*	*Miniopterus schreibersii*	G. Chiusazza	S	R	S	R	S	S	R	R	R	S	S	S	S	S	S	R	S	S	S
CH_N38	*Escherichia coli*	*Miniopterus schreibersii*	G. Chiusazza	S	R	S	S	S	S	S	S	S	S	S	S	S	R	R	S	S	S	S
CH_N39	*Escherichia coli*	*Miniopterus schreibersii*	G. Chiusazza	S	R	S	S	S	R	S	S	S	S	S	S	S	S	S	S	S	S	S
CH_N40	*Escherichia coli*	*Rhinolophus ferrumequinum*	G. Chiusazza	R	R	S	R	S	R	S	S	S	S	S	S	R	R	R	S	S	S	R
CH_N41	*Escherichia coli*	*Rhinolophus ferrumequinum*	G. Chiusazza	S	R	R	S	S	S	S	S	S	S	S	S	S	R	R	S	S	R	R
CH_N42	*Escherichia coli*	*Rhinolophus ferrumequinum*	G. Chiusazza	S	R	S	R	S	S	S	S	S	S	S	S	S	S	S	S	S	S	S
CH_N43	*Hafnia alvei*	*Miniopterus schreibersii*	G. Chiusazza	S	R	S	R	S	R	S	S	S	S	R	S	S	R	a	a	S	S	R
CH_N44	*Hafnia alvei*	*Miniopterus schreibersii*	G. Chiusazza	R	R	S	R	S	R	R	S	S	S	S	S	S	R	a	a	S	S	S
CH_N45	*Hafnia alvei*	*Miniopterus schreibersii*	G. Chiusazza	R	R	S	R	S	R	R	S	S	S	S	S	S	R	a	a	S	S	S
CH_N46	*Hafnia alvei*	*Miniopterus schreibersii*	G. Chiusazza	S	R	R	R	S	S	S	S	S	S	S	S	S	R	a	a	S	R	R

CH_N47	*Hafnia alvei*	*Rhinolophus ferrumequinum*	G. Chiusazza	R	R	R	R	S	S	S	S	S	S	S	S	S	S	S	a	S	R	R
CH_N48	*Klebsiella oxytoca*	*Rhinolophus hipposideros*	G. Chiusazza	S	R	R	S	S	S	S	S	S	S	S	S	S	R	a	a	S	R	R
CH_N49	*Klebsiella oxytoca*	*Rhinolophus hipposideros*	G. Chiusazza	S	S	S	R	S	S	S	S	S	S	S	S	S	R	a	a	S	R	R
CH_N50	*Klebsiella oxytoca*	*Rhinolophus hipposideros*	G. Chiusazza	S	R	R	R	S	S	S	S	S	S	S	S	S	R	a	a	S	S	S
CH_N51	*Klebsiella oxytoca*	*Rhinolophus hipposideros*	G. Chiusazza	S	S	S	S	S	S	S	S	S	S	S	S	S	R	a	a	S	S	S
CH_N52	*Klebsiella oxytoca*	*Miniopterus schreibersii*	G. Chiusazza	S	S	S	S	S	S	S	S	S	S	S	S	S	R	a	a	S	S	S
CH_N53	*Morganella morganii*	*Rhinolophus hipposideros*	G. Chiusazza	R	R	S	R	S	S	S	S	S	S	S	S	R	R	S	a	S	R	R
CH_N54	*Morganella morganii*	*Miniopterus schreibersii*	G. Chiusazza	S	S	S	S	S	S	S	S	S	R	S	S	S	R	a	a	S	S	R
CH_N55	*Morganella morganii*	*Miniopterus schreibersii*	G. Chiusazza	S	S	S	R	S	S	S	S	S	S	S	S	S	R	a	a	S	R	R
CH_N56	*Morganella morganii*	*Miniopterus schreibersii*	G. Chiusazza	S	I	S	S	S	S	S	S	S	S	S	S	S	R	a	a	S	S	S
CH_N57	*Morganella morganii*	*Miniopterus schreibersii*	G. Chiusazza	R	R	S	R	S	R	S	S	S	S	S	S	R	R	a	a	S	S	R
CH_N58	*Morganella morganii*	*Miniopterus schreibersii*	G. Chiusazza	S	R	S	R	S	R	S	S	S	R	S	S	S	S	I	a	S	R	R
CH_N59	*Morganella morganii*	*Rhinolophus ferrumequinum*	G. Chiusazza	S	R	R	R	S	I	R	R	S	S	S	S	S	R	S	S	S	R	R
CH_N60	*Morganella morganii*	*Rhinolophus ferrumequinum*	G. Chiusazza	S	R	R	R	S	R	R	R	S	S	S	S	S	R	S	S	S	R	R
CH_N61	*Morganella morganii*	*Rhinolophus ferrumequinum*	G. Chiusazza	S	S	S	S	S	S	S	S	S	I	S	S	S	R	a	a	S	S	S
CH_N62	*Morganella morganii*	*Rhinolophus ferrumequinum*	G. Chiusazza	S	S	S	S	S	S	S	S	S	S	S	S	S	R	a	a	S	S	S
CH_N63	*Proteus mirabilis*	*Miniopterus schreibersii*	G. Chiusazza	S	I	S	S	S	S	S	S	S	S	S	S	S	R	R	R	S	S	S
CH_N64	*Proteus mirabilis*	*Rhinolophus hipposideros*	G. Chiusazza	S	R	R	S	R	S	R	S	S	R	S	S	R	R	R	R	S	a	a
CH_N65	*Proteus vulgaris*	*Rhinolophus hipposideros*	G. Chiusazza	R	I	S	R	S	R	S	S	R	R	S	S	R	S	S	R	S	a	a
CH_N66	*Providencia rettgeri*	*Rhinolophus ferrumequinum*	G. Chiusazza	S	R	R	S	S	S	S	S	S	S	S	S	S	R	a	a	S	a	a
CH_N67	*Providencia rettgeri*	*Rhinolophus ferrumequinum*	G. Chiusazza	S	R	R	S	S	S	S	S	S	S	S	S	S	R	a	a	S	a	a
CH_N68	*Providencia rettgeri*	*Rhinolophus ferrumequinum*	G. Chiusazza	S	S	S	S	S	S	S	S	S	S	S	S	S	R	a	a	S	S	S
CH_N69	*Providencia rettgeri*	*Rhinolophus ferrumequinum*	G. Chiusazza	S	I	I	S	S	S	S	S	I	S	S	S	S	R	a	a	S	S	S
CH_N70	*Providencia rettgeri*	*Miniopterus schreibersii*	G. Chiusazza	S	R	R	S	S	S	S	S	S	R	S	S	S	R	a	a	S	S	a
CH_N71	*Providencia rettgeri*	*Rhinolophus hipposideros*	G. Chiusazza	S	R	R	R	S	S	S	S	S	S	S	S	S	R	S	a	S	a	a
CH_N72	*Providencia rettgeri*	*Rhinolophus hipposideros*	G. Chiusazza	S	R	R	S	S	S	S	S	S	S	S	S	S	R	a	a	S	a	I
CH_N73	*Providencia rettgeri*	*Rhinolophus hipposideros*	G. Chiusazza	S	S	S	S	S	S	S	S	S	S	S	S	S	R	a	a	S	S	S
CH_N74	*Rahnella aquatilis*	*Miniopterus schreibersii*	G. Chiusazza	S	S	R	I	R	R	S	S	R	S	R	S	R	R	R	R	R	S	S
CH_N76	*S. enterica* subsp. Arizonae	*Rhinolophus hipposideros*	G. Chiusazza	S	R	R	R	S	S	S	S	S	S	S	S	S	R	S	R	S	R	R
CH_N75	*S. enterica* subsp. Houtenae	*Rhinolophus hipposideros*	G. Chiusazza	R	S	R	R	S	S	S	S	S	S	S	S	S	S	I	S	S	R	R

CH_N78	*Serratia liquefaciens*	*Miniopterus schreibersii*	G. Chiusazza	S	R	R	R	S	R	R	S	S	S	S	S	S	R	S	R	S	S	S
CH_N77	*Serratia marcescens*	*Miniopterus schreibersii*	G. Chiusazza	S	R	R	R	S	S	R	R	S	S	S	S	S	R	S	S	S	S	R
CH_N79	*Serratia marcescens*	*Rhinolophus ferrumequinum*	G. Chiusazza	R	S	R	R	S	R	I	S	S	S	R	S	R	R	a	a	R	R	R
CH_N80	*Serratia marcescens*	*Rhinolophus hipposideros*	G. Chiusazza	R	R	R	R	S	S	R	R	S	I	S	S	S	R	S	S	S	R	I
CH_N81	*Serratia marcescens*	*Rhinolophus ferrumequinum*	G. Chiusazza	S	R	R	R	S	S	S	S	S	S	S	S	S	R	S	a	S	R	R
CH_N82	*Serratia spp.*	*Miniopterus schreibersii*	G. Chiusazza	S	R	R	R	S	S	R	R	R	S	S	S	R	R	R	R	S	S	S
CH_N83	*Serratia spp.*	*Miniopterus schreibersii*	G. Chiusazza	R	R	R	R	S	S	S	S	S	S	S	S	S	R	R	R	S	S	S
CAS_N1	*Achromobacter spp.*	*Rhinolophus euryale*	M. Castelluccio	R	S	R	S	S	S	S	S	I	S	S	S	I	R	R	S	S	I	S
CAS_N2	*Aeromonas hydrophila*	*Rhinolophus euryale*	M. Castelluccio	R	R	S	S	S	S	R	S	R	S	S	S	S	S	S	S	S	R	I
CAS_N3	*Aeromonas hydrophila*	*Rhinolophus euryale*	M. Castelluccio	R	R	R	S	S	S	S	S	S	S	S	S	S	S	S	S	S	S	S
CAS_N4	*Aeromonas sobria*	*Rhinolophus euryale*	M. Castelluccio	R	R	S	S	S	S	S	S	S	S	S	S	S	R	R	S	S	S	S
CAS_N5	*Escherichia coli*	*Rhinolophus ferrumequinum*	M. Castelluccio	R	S	R	R	S	R	R	S	S	S	S	S	R	R	R	R	S	S	S
CAS_N6	*Escherichia coli*	*Rhinolophus ferrumequinum*	M. Castelluccio	S	R	S	S	S	R	S	S	S	S	S	S	S	S	S	S	S	S	S
CAS_N7	*Escherichia coli*	*Rhinolophus ferrumequinum*	M. Castelluccio	S	S	S	S	S	S	S	S	S	S	S	S	S	R	R	R	S	S	S
CAS_N8	*Escherichia coli*	*Rhinolophus ferrumequinum*	M. Castelluccio	R	R	R	S	I	S	S	S	I	S	S	S	S	R	I	I	S	S	S
CAS_N9	*Escherichia coli*	*Rhinolophus ferrumequinum*	M. Castelluccio	S	R	S	R	S	R	S	S	S	S	S	S	S	S	S	S	S	S	S
CAS_N10	*Escherichia coli*	*Miniopterus schreibersii*	M. Castelluccio	S	S	R	S	I	I	S	S	S	S	S	S	S	R	R	R	I	S	S
CAS_N11	*Escherichia coli*	*Miniopterus schreibersii*	M. Castelluccio	S	S	S	R	S	S	S	S	S	S	S	S	S	R	R	R	S	S	S
CAS_N12	*Escherichia coli*	*Miniopterus schreibersii*	M. Castelluccio	R	R	R	S	I	I	S	S	S	S	S	S	S	S	S	R	S	S	S
CAS_N13	*Flavobacterium breve*	*Rhinolophus ferrumequinum*	M. Castelluccio	S	S	S	S	S	S	S	S	I	S	S	S	S	R	R	S	S	S	S
CAS_N14	*Hafnia alvei*	*Rhinolophus hipposideros*	M. Castelluccio	S	R	R	S	S	S	S	S	S	S	S	S	S	R	a	S	S	R	R
CAS_N15	*Moellerella winsconsensis*	*Rhinolophus hipposideros*	M. Castelluccio	R	R	R	R	S	R	S	S	S	S	S	S	R	S	S	S	S	S	R
CAS_N16	*Moellerella winsconsensis*	*Rhinolophus ferrumequinum*	M. Castelluccio	R	R	S	R	S	R	I	S	S	S	S	S	S	R	S	R	S	S	S
CAS_N17	*Moellerella winsconsensis*	*Rhinolophus ferrumequinum*	M. Castelluccio	S	S	S	R	S	S	S	S	S	S	S	S	S	R	R	R	S	S	S
CAS_N18	*Morganella morganii*	*Rhinolophus hipposideros*	M. Castelluccio	S	R	S	R	S	S	S	S	S	R	S	S	S	R	a	a	S	S	R
CAS_N19	*Morganella morganii*	*Rhinolophus hipposideros*	M. Castelluccio	S	R	R	R	S	S	S	S	S	S	S	S	S	R	a	a	S	S	S
CAS_N20	*Morganella morganii*	*Rhinolophus ferrumequinum*	M. Castelluccio	S	I	S	S	S	I	S	S	S	S	S	S	S	R	a	a	S	S	S
CAS_N21	*Pasteurella aerogenes*	*Rhinolophus ferrumequinum*	M. Castelluccio	S	R	R	S	S	S	S	S	S	R	S	S	S	R	R	R	R	R	R
CAS_N22	*Providencia rettgeri*	*Rhinolophus hipposideros*	M. Castelluccio	S	S	S	R	S	R	S	S	S	S	S	S	S	R	a	a	S	a	a
CAS_N23	*Pseudomonas alcaligenes*	*Rhinolophus hipposideros*	M. Castelluccio	S	R	R	R	S	S	R	R	S	S	S	S	S	R	S	S	S	S	R

CAS_N24	*Pseudomonas putida*	*Rhinolophus hipposideros*	M. Castelluccio	S	S	S	R	S	S	S	S	S	S	S	S	S	R	R	R	S	S	S
CAS_N25	*Serratia marcescens*	*Rhinolophus hipposideros*	M. Castelluccio	S	S	I	S	S	S	S	S	S	S	S	S	S	R	a	a	S	S	S
CAS_N26	*Serratia marcescens*	*Rhinolophus ferrumequinum*	M. Castelluccio	S	I	S	S	S	S	S	I	S	I	S	S	S	R	a	a	S	S	S

GEN: Gentamicin; STR: Streptomycin; TOB: Tobramycin; IMI: Imipenem; MERO: Meropenem; CTX: Cefotaxime; CAZ: Ceftazidime; CPT: Ceftazidime + clavulanic acid; CPT: Nalidixic acid; CHL: Chloramphenicol; CIP: Ciprofloxacin; ENRO: Enrofloxacin; AZT: Aztreonam; AMX: Amoxicillin; AMC: Amoxicillin + clavulanic acid; AMP: Ampicillin; SXT: Trimethoprim-Sulfamethoxazole; TET: Tetracyclin; DOX: Doxycyclin; R: Resistant; I: Intermediate Susceptibility; S: Susceptible; a: Intrinsic Resistance.

Table S4. Gram-positive from bats: bacterial species identification, bats species, sampling site, and susceptibility data.

Isolate ID	Bacterial Species	Bats Species	Sampling Site	GEN	TOB	IMI	MERO	CAZ	FEP	CPT	ENR	VA	LIN	ERY	AML	AMP	OXA	AMC	SAM	TCC	MH	TET
CAP1	*Bacillus licheniformis*	*Miniopterus schreibersii*	G. Caprara	R	R	S	S	S	S	R	S	S	R	S	S	S	S	S	S	S	S	S
CAP2	*Bacillus licheniformis*	*Miniopterus schreibersii*	G. Caprara	S	S	S	I	R	S	S	S	S	S	S	S	S	S	R	S	R	S	S
CAP6	*Bacillus licheniformis*	*Rhinolophus ferrumequinum*	G. Caprara	S	R	S	S	S	R	S	S	S	S	S	R	R	R	S	R	R	S	S
CAP8	*Bacillus licheniformis*	*Miniopterus schreibersii*	G. Caprara	S	R	S	S	S	S	R	S	S	S	S	R	S	R	S	S	S	S	S
CAP5	*Enterococcus faecalis*	*Miniopterus schreibersii*	G. Caprara	S	S	R	S	a	a	a	S	R	R	a	S	S	R	S	S	S	S	S
CAP10	*Enterococcus faecalis*	*Miniopterus schreibersii*	G. Caprara	S	S	S	R	S	a	a	S	S	R	S	S	R	R	S	S	S	S	S
CAP3	*Staphylococcus epidermidis*	*Miniopterus schreibersii*	G. Caprara	R	R	S	S	S	S	S	R	R	R	R	S	R	R	R	S	S	S	S
CAP7	*Staphylococcus epidermidis*	*Miniopterus schreibersii*	G. Caprara	S	S	S	S	S	S	S	S	R	S	R	S	R	R	S	S	R	S	S
CAP4	*Staphylococcus warneri*	*Rhinolophus ferrumequinum*	G. Caprara	R	R	S	S	a	S	R	S	R	S	R	R	R	R	S	S	S	S	S
CAP9	*Staphylococcus warneri*	*Rhinolophus euryale*	G. Caprara	R	R	I	R	a	R	S	S	S	R	R	R	S	R	R	S	S	S	S
CH22	*Bacillus cereus*	*Miniopterus schreibersii*	G. Chiusazza	S	S	R	S	R	S	S	S	S	S	S	R	R	S	R	S	S	S	S
CH3	*Bacillus licheniformis*	*Rhinolophus ferrumequinum*	G. Chiusazza	S	R	S	S	S	R	R	S	R	S	R	S	R	R	R	S	S	S	S
CH4	*Bacillus licheniformis*	*Miniopterus schreibersii*	G. Chiusazza	R	R	S	S	R	S	R	S	S	R	S	R	R	R	S	S	S	S	S
CH11	*Bacillus licheniformis*	*Rhinolophus hipposideros*	G. Chiusazza	S	S	S	R	R	S	I	S	S	S	S	S	S	S	S	S	I	S	S
CH6	*Bacillus safensis*	*Miniopterus schreibersii*	G. Chiusazza	S	S	S	S	R	R	R	S	R	R	R	R	R	R	S	S	S	S	S
CH5	*Bacillus velezensis*	*Miniopterus schreibersii*	G. Chiusazza	S	S	S	S	S	S	R	S	S	R	I	S	S	R	S	S	S	S	S
CH7	*Cytobacillus horneckiae*	*Miniopterus schreibersii*	G. Chiusazza	S	S	R	R	R	S	S	S	S	R	S	R	S	R	S	S	S	S	S
CH17	*Enterococcus casseliflavus*	*Rhinolophus hipposideros*	G. Chiusazza	R	R	R	S	R	R	R	R	R	R	a	S	S	S	S	S	S	S	S
CH23	*Enterococcus faecalis*	*Miniopterus schreibersii*	G. Chiusazza	a	R	R	S	a	a	a	R	R	R	a	S	R	S	R	S	S	S	S
CH9	*Staphylococcus capitis*	*Rhinolophus ferrumequinum*	G. Chiusazza	S	R	R	S	a	R	S	S	R	R	S	R	R	R	R	S	R	S	S

CH2	*Staphylococcus cohnii*	*Miniopterus schreibersii*	G. Chiusazza	R	S	S	S	S	R	R	S	I	I	S	S	S	S	S	S	S	S	S
CH19	*Staphylococcus cohnii*	*Rhinolophus hipposideros*	G. Chiusazza	R	S	S	S	a	S	R	S	R	R	R	S	R	S	S	S	S	S	S
CH12	*Staphylococcus epidermidis*	*Rhinolophus ferrumequinum*	G. Chiusazza	S	S	I	R	a	S	S	S	S	R	S	S	R	R	S	S	S	S	S
CH13	*Staphylococcus sciuri*	*Rhinolophus ferrumequinum*	G. Chiusazza	S	S	I	R	a	S	S	S	R	S	S	R	S	R	S	S	S	R	S
CH24	*Staphylococcus sciuri*	*Rhinolophus hipposideros*	G. Chiusazza	S	S	S	R	S	R	S	S	R	R	R	R	S	R	S	S	S	S	S
CH15	*Staphylococcus simulans*	*Miniopterus schreibersii*	G. Chiusazza	S	S	S	R	a	S	R	S	S	I	I	S	S	S	S	S	S	S	S
CH12	*Staphylococcus warneri*	*Rhinolophus ferrumequinum*	G. Chiusazza	R	R	S	R	a	S	S	R	S	R	R	S	S	R	R	S	S	S	S
CH14	*Staphylococcus warneri*	*Miniopterus schreibersii*	G. Chiusazza	R	R	R	S	a	R	S	R	S	R	R	S	S	R	R	S	S	S	S
CH16	*Staphylococcus warneri*	*Rhinolophus hipposideros*	G. Chiusazza	R	R	S	S	a	S	S	R	R	R	R	S	R	R	R	S	S	S	S
CH1	*Staphylococcus xylosus*	*Miniopterus schreibersii*	G. Chiusazza	S	R	R	R	a	S	S	S	R	R	R	S	S	R	R	S	R	S	S
CH8	*Staphylococcus xylosus*	*Miniopterus schreibersii*	G. Chiusazza	S	S	S	I	S	I	S	S	R	R	R	R	S	R	R	S	S	S	S
CH10	*Staphylococcus xylosus*	*Miniopterus schreibersii*	G. Chiusazza	R	R	R	S	a	R	S	R	R	R	R	S	S	S	S	S	S	S	S
CH18	*Staphylococcus xylosus*	*Rhinolophus ferrumequinum*	G. Chiusazza	R	S	R	S	a	R	S	S	R	R	R	S	R	R	S	S	S	S	S
CH20	*Staphylococcus xylosus*	*Miniopterus schreibersii*	G. Chiusazza	R	R	R	S	a	S	S	S	R	S	S	S	R	R	S	S	R	S	S
CH21	*Staphylococcus xylosus*	*Miniopterus schreibersii*	G. Chiusazza	R	S	R	S	a	R	S	R	R	R	R	S	S	S	S	S	S	S	S
CAS1	*Bacillus atrophaeus*	*Rhinolophus ferrumequinum*	M. Castelluccio	S	S	S	R	R	S	S	S	S	R	S	S	S	R	S	S	S	S	S
CAS2	*Bacillus licheniformis*	*Rhinolophus ferrumequinum*	M. Castelluccio	R	R	R	S	R	S	S	S	R	R	R	S	R	R	S	S	S	I	R
CAS3	*Bacillus licheniformis*	*Rhinolophus hipposideros*	M. Castelluccio	R	R	S	S	R	S	R	S	S	R	S	R	R	R	S	S	S	S	S
CAS4	*Bacillus licheniformis*	*Rhinolophus ferrumequinum*	M. Castelluccio	S	R	S	S	R	R	S	S	S	R	S	S	S	R	S	S	R	S	S
CAS5	*Bacillus licheniformis*	*Rhinolophus ferrumequinum*	M. Castelluccio	S	S	S	S	S	S	S	S	R	R	R	R	R	R	S	S	S	I	R
CAS6	*Bacillus licheniformis*	*Rhinolophus hipposideros*	M. Castelluccio	R	R	S	S	R	S	R	S	S	R	S	R	R	R	S	S	S	S	S
CAS7	*Bacillus licheniformis*	*Rhinolophus ferrumequinum*	M. Castelluccio	R	R	S	S	R	S	R	S	S	R	I	R	R	R	S	S	S	S	S
CAS8	*Bacillus subtilis*	*Rhinolophus ferrumequinum*	M. Castelluccio	R	R	R	S	R	R	S	S	R	R	S	S	S	R	S	S	S	S	S

GEN: Gentamicin; TOB: Tobramycin; IMI: Imipenem; MERO: Meropenem; CAZ: Ceftazidime; FEP: Cefepime; CPT: Ceftaroline; ENR: Enrofloxacin; VAN: Vancomycin; LIN: Lincomycin; ERY: Erythromycin; AMX: Amoxicillin; AMP: Ampicillin; OXA: Oxacillin; AMC: Amoxicillin + clavulanic acid; SAM: Ampicillin + sulbactam; TCC: Ticarcillin + clavulanic acid; MH: Minocycline; TET: Tetracycline; R: Resistant; I: Intermediate Susceptibility; S: Susceptible; a: Intrinsic Resistance

Table S5. MDR patterns of bacterial isolates from bats: host species and cave of origin.

Isolate ID	Bacterial Species	Bats Species	Sampling Site	MDR Profiles
B12	*Hafnia alvei*	*Miniopterus schreibersii*	G. Burrò	AMI, CAR, CEF, CLO, FLU, TET
B11	*Escherichia coli*	*Rhinolophus euryale*	G. Burrò	AMI, CEF, CAR, CHI, FLU, MON, PEN
B10	*Escherichia coli*	*Rhinolophus ferrumequinum*	G. Burrò	AMI, CEF, CAR, CHI, FLU, MON, PEN, TET, POL
B5	*Citrobacter freundii*	*Rhinolophus euryale*	G. Burrò	AMI, CEF, CAR, CHI, TET
B14	*Morganella morganii*	*Myotis myotis*	G. Burrò	AMI, CEF, CAR, MON, PEN, SUL, TET, CLO
B9	*Enterobacter cloacae*	*Miniopterus schreibersii*	G. Burrò	AMI, CEF, CHI, MON, TET
B2	*Alcaligenes faecalis*	*Myotis myotis*	G. Burrò	AMI, CEF, PEN
B7	*Enterobacter cloacae*	*Rhinolophus euryale*	G. Burrò	AMI, CLO, SUL, TET
B3	*Citrobacter diversus*	*Myotis myotis*	G. Burrò	AMI, PEN, TET
B17	*Providencia rettgeri*	*Rhinolophus ferrumequinum*	G. Burrò	CAR, CLO, PEN, TET
CAP_N20	*Pseudomonas aeruginosa*	*Miniopterus schreibersii*	G. Caprara	AMI, CAR, CHI, CLO, TET
CAP_N11	*Enterobacter asburiae*	*Miniopterus schreibersii*	G. Caprara	AMI, CAR, PEN
CAP_N14	*Enterobacter hormaechei*	*Rhinolophus euryale*	G. Caprara	AMI, CAR, PEN
CAP_N19	*Pseudocitrobacter faecalis*	*Miniopterus schreibersii*	G. Caprara	AMI, CAR, TET, POL
CAP_N10	*Citrobacter spp.*	*Rhinolophus euryale*	G. Caprara	AMI, CEF, CAR, CHI, MON, PEN
CAP_N22	*Serratia odorifera*	*Rhinolophus ferrumequinum*	G. Caprara	AMI, CEF, TET
CAP_N12	*Enterobacter cloacae*	*Miniopterus schreibersii*	G. Caprara	AMI, CLO, SUL, TET
CAP_N16	*Escherichia coli*	*Miniopterus schreibersii*	G. Caprara	AMI, PEN, SUL, TET
CAP_N18	*Morganella morganii*	*Rhinolophus ferrumequinum*	G. Caprara	AMI, PEN, TET
CAP_N21	*Serratia marcescens*	*Rhinolophus ferrumequinum*	G. Caprara	AMI, PEN, TET, POL
CAP_N2	*Citrobacter braakii*	*Rhinolophus euryale*	G. Caprara	AMI. CEF, CAR, PEN, TET
CAP1	*Bacillus licheniformis*	*Miniopterus schreibersii*	G. Caprara	AMI, CEF, LIN
CAP6	*Bacillus licheniformis*	*Rhinolophus ferrumequinum*	G. Caprara	AMI, CEF, PEN
CAP8	*Bacillus licheniformis*	*Miniopterus schreibersii*	G. Caprara	AMI, CEF, PEN
CAP5	*Enterococcus faecalis*	*Miniopterus schreibersii*	G. Caprara	CAR, GLI, LIN, PEN
CAP10	*Enterococcus faecalis*	*Miniopterus schreibersii*	G. Caprara	CAR, LIN, PEN
CAP3	*Staphylococcus epidermidis*	*Miniopterus schreibersii*	G. Caprara	AMI, FLU, GLI, LIN, MAC, PEN
CAP7	*Staphylococcus epidermidis*	*Miniopterus schreibersii*	G. Caprara	GLI, MAC, PEN
CAP4	*Staphylococcus warneri*	*Rhinolophus ferrumequinum*	G. Caprara	AMI, CEF, GLI, MAC, PEN

CAP9	*Staphylococcus warneri*	*Rhinolophus euryale*	G. Caprara	AMI, CAR, CEF, FLU, LIN, MAC, PEN
CH_N6	*Citrobacter freundii*	*Rhinolophus ferrumequinum*	G. Chiusazza	AMI, CAR, CEF
CH_N65	*Proteus vulgaris*	*Rhinolophus hipposideros*	G. Chiusazza	AMI, CAR, CEF, CHI, CLO, MON, PEN, TET
CH_N74	*Rahnella aquatilis*	*Miniopterus schreibersii*	G. Chiusazza	AMI, CAR, CEF, CHI, FLU, MON, PEN, SUL
CH_N82	*Serratia spp.*	*Miniopterus schreibersii*	G. Chiusazza	AMI, CAR, CEF, CHI, MON, PEN
CH_N37	*Escherichia coli*	*Miniopterus schreibersii*	G. Chiusazza	AMI, CAR, CEF, CHI, PEN
CH_N4	*Citrobacter freundii*	*Miniopterus schreibersii*	G. Chiusazza	AMI, CAR, CEF, CHI, TET
CH_N64	*Proteus mirabilis*	*Rhinolophus hipposideros*	G. Chiusazza	AMI, CAR, CEF, COL, MON, PEN
CH_N79	*Serratia marcescens*	*Rhinolophus ferrumequinum*	G. Chiusazza	AMI, CAR, CEF, FLU, MON, PEN, SUL, TET
CH_N44	*Hafnia alvei*	*Miniopterus schreibersii*	G. Chiusazza	AMI, CAR, CEF, PEN
CH_N45	*Hafnia alvei*	*Miniopterus schreibersii*	G. Chiusazza	AMI, CAR, CEF, PEN
CH_N36	*Escherichia coli*	*Rhinolophus ferrumequinum*	G. Chiusazza	AMI, CAR, CEF, PEN, TET
CH_N80	*Serratia marcescens*	*Rhinolophus hipposideros*	G. Chiusazza	AMI, CAR, CEF, PEN, TET
CH_N35	*Escherichia coli*	*Rhinolophus hipposideros*	G. Chiusazza	AMI, CAR, CEF, PEN, TET
CH_N75	*S. enterica subsp. Houtenae*	*Rhinolophus hipposideros*	G. Chiusazza	AMI, CAR, CEF, TET
CH_N3	*Citrobacter freundii*	*Rhinolophus hipposideros*	G. Chiusazza	AMI, CAR, CEF, TET
CH_N78	*Serratia liquefaciens*	*Miniopterus schreibersii*	G. Chiusazza	AMI, CAR, CEF, TET
CH_N19	*Enterobacter asburiae*	*Miniopterus schreibersii*	G. Chiusazza	AMI, CAR, CHI, MON, PEN, TET
CH_N53	*Morganella morganii*	*Rhinolophus hipposideros*	G. Chiusazza	AMI, CAR, MON, PEN, TET
CH_N50	*Klebsiella oxytoca*	*Rhinolophus hipposideros*	G. Chiusazza	AMI, CAR, PEN
CH_N83	*Serratia spp.*	*Miniopterus schreibersii*	G. Chiusazza	AMI, CAR, PEN
CH_N47	*Hafnia alvei*	*Rhinolophus ferrumequinum*	G. Chiusazza	AMI, CAR, PEN, TET
CH_N71	*Providencia rettgeri*	*Rhinolophus hipposideros*	G. Chiusazza	AMI, CAR, PEN, TET
CH_N46	*Hafnia alvei*	*Miniopterus schreibersii*	G. Chiusazza	AMI, CAR, PEN, TET
CH_N76	*S. enterica subsp. Arizonae*	*Rhinolophus hipposideros*	G. Chiusazza	AMI, CAR, PEN, TET
CH_N81	*Serratia marcescens*	*Rhinolophus ferrumequinum*	G. Chiusazza	AMI, CAR, PEN, TET
CH_N12	*Citrobacter spp.*	*Miniopterus schreibersii*	G. Chiusazza	AMI, CEF, CAR, CHI, MON, PEN
CH_N18	*Enterobacter asburiae*	*Miniopterus schreibersii*	G. Chiusazza	AMI, CEF, CAR, CHI, MON, PEN, SUL, TET
CH_N33	*Escherichia coli*	*Rhinolophus hipposideros*	G. Chiusazza	AMI, CEF, CAR, CHI, MON, PEN, TET
CH_N10	*Citrobacter gillenii*	*Rhinolophus ferrumequinum*	G. Chiusazza	AMI, CEF, CAR, CHI, PEN
CH_N11	*Citrobacter gillenii*	*Rhinolophus ferrumequinum*	G. Chiusazza	AMI, CEF, CAR, CHI, PEN
CH_N58	*Morganella morganii*	*Miniopterus schreibersii*	G. Chiusazza	AMI, CEF, CAR, CLO, TET
CH_N43	*Hafnia alvei*	*Miniopterus schreibersii*	G. Chiusazza	AMI, CEF, CAR, FLU, PEN, TET
CH_N34	*Escherichia coli*	*Rhinolophus hipposideros*	G. Chiusazza	AMI, CEF, CAR, MON, PEN, TET
CH_N40	*Escherichia coli*	*Rhinolophus ferrumequinum*	G. Chiusazza	AMI, CEF, CAR, MON, PEN, TET
CH_N57	*Morganella morganii*	*Miniopterus schreibersii*	G. Chiusazza	AMI, CEF, CAR, MON, PEN, TET
CH_N5	*Citrobacter freundii*	*Miniopterus schreibersii*	G. Chiusazza	AMI, CEF, CHI
CH_N30	*Enterobacter hormaechei*	*Rhinolophus hipposideros*	G. Chiusazza	AMI, CEF, TET

CH_N70	*Providencia rettgeri*	*Miniopterus schreibersii*	G. Chiusazza	AMI, CLO, PEN, TET
CH_N32	*Enterobacter spp.*	*Rhinolophus hipposideros*	G. Chiusazza	AMI, PEN, POL
CH_N20	*Enterobacter cancerogenus*	*Rhinolophus hipposideros*	G. Chiusazza	AMI, PEN, SUL, TET, CLO
CH_N66	*Providencia rettgeri*	*Rhinolophus ferrumequinum*	G. Chiusazza	AMI, PEN, TET
CH_N14	*Citrobacter spp.*	*Miniopterus schreibersii*	G. Chiusazza	AMI, PEN, TET
CH_N31	*Enterobacter hormaechei*	*Rhinolophus hipposideros*	G. Chiusazza	AMI, PEN, TET
CH_N48	*Klebsiella oxytoca*	*Rhinolophus hipposideros*	G. Chiusazza	AMI, PEN, TET
CH_N67	*Providencia rettgeri*	*Rhinolophus ferrumequinum*	G. Chiusazza	AMI, PEN, TET
CH_N41	*Escherichia coli*	*Rhinolophus ferrumequinum*	G. Chiusazza	AMI, PEN, TET
CH_N72	*Providencia rettgeri*	*Rhinolophus hipposideros*	G. Chiusazza	AMI, PEN, TET
CH_N77	*Serratia marcescens*	*Miniopterus schreibersii*	G. Chiusazza	AMI. CAR, CEF, PEN, TET
CH_N13	*Citrobacter spp.*	*Miniopterus schreibersii*	G. Chiusazza	AMI. CEF, CAR, PEN, TET
CH_N59	*Morganella morganii*	*Rhinolophus ferrumequinum*	G. Chiusazza	AMI. CEF, CAR, PEN, TET
CH_N60	*Morganella morganii*	*Rhinolophus ferrumequinum*	G. Chiusazza	AMI. CEF, CAR, PEN, TET
CH_N49	*Klebsiella oxytoca*	*Rhinolophus hipposideros*	G. Chiusazza	CAR, PEN, TET
CH_N55	*Morganella morganii*	*Miniopterus schreibersii*	G. Chiusazza	CAR, PEN, TET
CH_N54	*Morganella morganii*	*Miniopterus schreibersii*	G. Chiusazza	PEN, TET, CLO
CH_N73	*Providencia rettgeri*	*Rhinolophus hipposideros*	G. Chiusazza	PEN, TET, POL
CH22	*Bacillus cereus*	*Miniopterus schreibersii*	G. Chiusazza	CAR, CEF, PEN
CH3	*Bacillus licheniformis*	*Rhinolophus ferrumequinum*	G. Chiusazza	AMI, CEF, GLI, MAC, PEN
CH4	*Bacillus licheniformis*	*Miniopterus schreibersii*	G. Chiusazza	AMI, CEF, LIN, PEN
CH6	*Bacillus safensis*	*Miniopterus schreibersii*	G. Chiusazza	CEF, GLI, LIN, MAC, PEN
CH5	*Bacillus velezensis*	*Miniopterus schreibersii*	G. Chiusazza	CEF, LIN, PEN
CH7	*Cytobacillus horneckiae*	*Miniopterus schreibersii*	G. Chiusazza	CAR, CEF, LIN, PEN
CH17	*Enterococcus casseliflavus*	*Rhinolophus hipposideros*	G. Chiusazza	AMI, CEF, FLU, GLI, LIN, PEN
CH23	*Enterococcus faecalis*	*Miniopterus schreibersii*	G. Chiusazza	CAR, FLU, GLI, LIN, PEN
CH9	*Staphylococcus capitis*	*Rhinolophus ferrumequinum*	G. Chiusazza	AMI, CAR, CEF, GLI, LIN, PEN
CH19	*Staphylococcus cohnii*	*Rhinolophus hipposideros*	G. Chiusazza	AMI, CEF, GLI, LIN, MAC, PEN
CH12	*Staphylococcus epidermidis*	*Rhinolophus ferrumequinum*	G. Chiusazza	CAR, LIN, PEN
CH13	*Staphylococcus sciuri*	*Rhinolophus ferrumequinum*	G. Chiusazza	CAR, GLI, LIN, MAC, PEN, TET
CH24	*Staphylococcus sciuri*	*Rhinolophus hipposideros*	G. Chiusazza	CEF, GLI, LIN, MAC, PEN
CH12	*Staphylococcus warneri*	*Rhinolophus ferrumequinum*	G. Chiusazza	AMI, CAR, FLU, LIN, MAC, PEN
CH14	*Staphylococcus warneri*	*Miniopterus schreibersii*	G. Chiusazza	AMI, CAR, CEF, FLU, LIN, MAC, PEN
CH16	*Staphylococcus warneri*	*Rhinolophus hipposideros*	G. Chiusazza	AMI, CAR, GLI, LIN, MAC, PEN
CH1	*Staphylococcus xylosus*	*Miniopterus schreibersii*	G. Chiusazza	AMI, CAR, GLI, LIN, MAC, PEN
CH8	*Staphylococcus xylosus*	*Miniopterus schreibersii*	G. Chiusazza	GLI, LIN, MAC, PEN
CH10	*Staphylococcus xylosus*	*Miniopterus schreibersii*	G. Chiusazza	AMI, CAR, CEF, FLU, GLI, LIN, MAC, PEN
CH18	*Staphylococcus xylosus*	*Rhinolophus ferrumequinum*	G. Chiusazza	AMI, CAR, CEF, GLI, LIN, MAC, PEN

CH20	*Staphylococcus xylosus*	*Miniopterus schreibersii*	G. Chiusazza	AMI, CAR, GLI, LIN, MAC, PEN
CH21	*Staphylococcus xylosus*	*Miniopterus schreibersii*	G. Chiusazza	AMI, CAR, CEF, FLU, GLI, LIN, MAC, PEN
CAS_N9	*Escherichia coli*	*Rhinolophus ferrumequinum*	M. Castelluccio	AMI, CAR, CEF
CAS_N5	*Escherichia coli*	*Rhinolophus ferrumequinum*	M. Castelluccio	AMI, CAR, CEF, MON, PEN
CAS_N16	*Moellerella winsconsensis*	*Rhinolophus ferrumequinum*	M. Castelluccio	AMI, CAR, CEF, PEN
CAS_N18	*Morganella morganii*	*Rhinolophus hipposideros*	M. Castelluccio	AMI, CAR, CLO, PEN, TET
CAS_N19	*Morganella morganii*	*Rhinolophus hipposideros*	M. Castelluccio	AMI, CAR, PEN
CAS_N15	*Moellerella winsconsensis*	*Rhinolophus hipposideros*	M. Castelluccio	AMI, CEF, CAR, MON, TET
CAS_N2	*Aeromonas hydrophila*	*Rhinolophus euryale*	M. Castelluccio	AMI, CEF, CHI, MON, TET
CAS_N21	*Pasteurella aerogenes*	*Rhinolophus ferrumequinum*	M. Castelluccio	AMI, CLO, PEN, SUL, TET
CAS_N14	*Hafnia alvei*	*Rhinolophus hipposideros*	M. Castelluccio	AMI, PEN, TET
CAS_N23	*Pseudomonas alcaligenes*	*Rhinolophus hipposideros*	M. Castelluccio	AMI,CAR, CEF, PEN, TET
CAS_N22	*Providencia rettgeri*	*Rhinolophus hipposideros*	M. Castelluccio	CEF, CAR, PEN, TET, POL
CAS1	*Bacillus atrophaeus*	*Rhinolophus ferrumequinum*	M. Castelluccio	CAR, CEF, LIN, PEN
CAS2	*Bacillus licheniformis*	*Rhinolophus ferrumequinum*	M. Castelluccio	AMI, CAR, GLI, LIN, MAC, PEN, TET
CAS3	*Bacillus licheniformis*	*Rhinolophus hipposideros*	M. Castelluccio	AMI, CEF, LIN, MAC, PEN
CAS4	*Bacillus licheniformis*	*Rhinolophus ferrumequinum*	M. Castelluccio	AMI, CEF, LIN, PEN
CAS5	*Bacillus licheniformis*	*Rhinolophus ferrumequinum*	M. Castelluccio	GLI, LIN, MAC, PEN, TET
CAS6	*Bacillus licheniformis*	*Rhinolophus hipposideros*	M. Castelluccio	AMI, CEF, LIN, PEN
CAS7	*Bacillus licheniformis*	*Rhinolophus ferrumequinum*	M. Castelluccio	AMI, CEF, LIN, PEN
CAS8	*Bacillus subtilis*	*Rhinolophus ferrumequinum*	M. Castelluccio	AMI, CEF, CAR, GLI, LIN

AMI = Aminoglycosides; CEF = cephalosporins; CAR = carbapenems; CHI = quinolones; CLO = chloramphenicol; FLU = fluoroquinolones; GLI = glycopeptides; LIN = lincosamides; MAC = macrolides; MON = monobactams; PEN = penicillins; SUL = sulphonamides; TET = tetracyclines.
